# Relationships between Anxiety, Emotional Intelligence, and Motivational Climate among Adolescent Football Players

**DOI:** 10.3390/sports7020034

**Published:** 2019-02-01

**Authors:** Manuel Castro-Sánchez, Félix Zurita-Ortega, José Luis Ubago-Jiménez, Gabriel González-Valero, Eduardo García-Mármol, Ramón Chacón-Cuberos

**Affiliations:** 1 Department of Didactics of Musical, Plastic and Corporal Expression, University of Granada, Granada 18071, Spain; manuelcs@ugr.es (M.C.-S.); felixzo@ugr.es (F.Z.-O.); jlubago@ugr.es (J.L.U.-J.); ggvalero@ugr.es (G.G.-V.); 2 Department of Physical Education and Sports, University of Granada, Granada 18071, Spain; eduardogarcia@ugr.es; 3 Department of Research and Diagnosis Methods in Education, University of Granada, Granada 18071, Spain

**Keywords:** anxiety, emotional intelligence, motivational climate, football

## Abstract

Background: Emotional and motivational factors are fundamental in the context of sport, as they directly relate to sports performance and anxiety. Methods: The present study aimed to analyze the relationships between motivational climate (MC), emotional intelligence (EI), and anxiety within a sample of footballers playing at a low level. The sample was composed of 282 registered football players aged between 16 and 18 years old (16.96 ± 0.77), playing in the lower tier in the province of Jaen (Spain). Data were self-reported, with participants responding to the Perceived Motivational Climate in Sport Questionnaire (PMCSQ-2), the Schutte Self-Report Inventory (SSRI), and the State Trait Anxiety Inventory (STAI). Results: The results showed that footballers who reported higher levels of state anxiety and trait anxiety also demonstrated lower EI and more negatively perceived and regulated their emotions. Moreover, an ego-oriented climate was associated with higher levels of anxiety, while a task-oriented climate was related to lower levels of anxiety and higher levels of EI. No relationship was identified between the emotional aspects of young footballers and holding a motivational orientation toward an ego climate. Conclusions: Football players who more greatly perceived a task-oriented climate had higher EI and usually reported lower levels of anxiety related to sport performance. It is therefore important to promote intrinsic motivations and develop the capacity of footballers to regulate their own emotions.

## 1. Introduction

One of the most popular sports currently practiced by young people is football [1,2]. Football is a collective sport that involves two teams competing in opposition within a limited space. The aim of the sport is to obtain possession of the ball and introduce it into the opponent’s goal [3]. It is a highly dynamic sport in which social, psychological, and technical-tactical factors interact implicitly [4,5]. The sport is characterized as being one of the most popular sports worldwide according to the number of people participating, particularly in continents such as Europe and South America. It attracts a large number of followers due to the combination of simple technical movements, which provoke strong emotional responses in spectators [6,7]. In addition, it is worth noting that football is becoming a great attraction for new generations in continents such as North America and Asia, where it is growing in popularity [8,9].

Psychological aspects have played a very important role throughout history in the sporting context [10], but it was not until about twenty years ago that they began to gain great relevance. It is currently inconceivable that a sports club would not count on a psychology specialist in order to maximize performance in training and competition [11]. Motivational processes and mental aspects such as anxiety and emotional intelligence (EI) have been studied under the lens of sport psychology in order to understand how they relate to sport performance [12,13]. This presents an important context of interest in which interventions can be developed with the aim of inducing appropriate motivational states from an early age [14].

Motivation is one of the most studied psychological factors in sport psychology literature and by researchers of sport performance. In this context, an important focus of interest relates to achievement goals, which are influenced by various factors with great potential for explaining human behavior [15,16]. Motivation is also considered a phenomenon that integrates interrelated emotional, biological, social, and cognitive aspects, and produces observable behaviors [13,17]. Due to its importance, contemporary studies of motivation in the sports environment have been framed according to the achievement goal theory [18]. This is based on the idea that people establish objectives according to the perceptions they have regarding their own skills [19]. Within this framework, the concept of motivational climate (MC) is presented as a set of implicit and explicit signals that are perceived from the environment and through which the criteria of failure and success are defined [20,21]. In the sports field, coaches and technical teams can create environments oriented toward task- (mastery) or toward ego-based climates (performance), depending on the desired purpose [22]. As a result, coaches assume an important role in the sports context, as their different actions determine whether or not the desired environment is achieved. If the aim is enjoyment and maintaining regular engagement in sports, personal improvement and directing effort toward achieving mastery will be promoted [23]. On the other hand, when a competitive climate is desired, intrateam rivalry and environments oriented toward performance will be promoted [24]. As a result, athletes will adopt a motivational climate associated with either intrinsic or extrinsic motivations [25].

Anxiety is another aspect that must be addressed within sports psychology. Anxiety is defined as a negative psycho-emotional state that results when fear of events, which are not always identifiable, manifests as an exaggerated response where nervousness and worry predominate [26,27]. An anxious response in athletes can be identified by observing biological and cognitive markers, such as breathing, learning processes, and conditioning [28,29]. Thus, it is important that athletes focus on the activity they are engaged in, paying attention to their skills, feelings, and the goals that they want to achieve. Athletes must develop their capacities of self-control and self-confidence in order to regulate their states of alertness produced by anxiety, isolating themselves from the stressors around them [30,31]. Anxiety as a mental health problem has been studied in the sports field by several researchers. Research has specifically focused on high-level sport performance, given that these athletes are exposed to situations of greater stress and anxiety [32,33,34]. There is therefore a need to study these problems within populations that engage in lower-level physical activity in order to teach and help control the different psychological processes experienced throughout athletes’ careers [35,36].

Finally, EI is essential within the sports environment [37], as it facilitates psychological well-being by reducing the negative sensations related to anxiety and stress that can result from the demands of sport [38]. This concept has been described by Goleman [39] as the capacity of individuals to understand their own feelings and those of others, as well as the ability to manage these feelings adequately. There have been several authors who regard EI as an aspect of great relevance within the sports context due to the effect this construct has on emotional regulation in different situations [40,41] and its influence on the ability to make decisions in stressful situations [42]. Recent research has highlighted the need to develop emotional regulation because of its direct association with goals and ultimately performance success. Likewise, emotional control is essential for achieving effective control of skills, abilities, and emotional capabilities in sport [43,44].

When considering football, the coach’s role extends beyond determining the technical-tactical preparation required to reach a higher level of performance. Psychological factors are especially relevant due to the aforementioned relationships between sports performance and control of anxiety, emotional regulation, and aspects related to motivation [28,45,46,47]. Thus, these aspects must be considered when planning training sessions. The coach plays a fundamental role in developing certain motivational climates at professional and nonprofessional levels of sport. It is important for them to consider that certain achievement goals may favor states of anxiety that are associated with impaired sport performance and negative emotions such as frustration [48]. Task-oriented climates that promote intrinsic motivations could act to prevent negative emotional states, providing an excellent resource for developing positive emotions and decreasing stress [24,49]. Thus, coaches and other members of the technical team are essential in supporting the regulation and use of positive emotional states: For instance, promoting high levels of EI with a view toward encouraging intrinsic motivations and reducing anxiety [50].

Considering what is already known about the role of stress and anxiety states during sport practice, it can be concluded that negative states are compounded by a low capacity for emotional regulation, as well as excessively competitive environments associated with ego-oriented motivational climates. The present study addressed the following research question with the aim of generating strategies to establish motivational climates that support the development of positive EI and reduced anxiety: Are motivational climate and emotional intelligence related to the anxiety levels of young low-level footballers?

Given the issues raised above and the need to identify efficient responses, the following hypotheses were proposed for the present study:

- Hypothesis 1 (H1). Football players with higher levels of emotional intelligence will also show lower levels of state anxiety and lower levels of trait anxiety.
- Hypothesis 2 (H2). Football players who report perceiving a task-oriented motivational climate to a greater extent will also demonstrate lower levels of anxiety. Footballers who report perceiving an ego-oriented climate to a greater extent will demonstrate higher levels of state and trait anxiety.
- Hypothesis 3 (H3). There will be a positive relationship between task-oriented motivational climate and the different dimensions of emotional intelligence. This relationship will be negative for ego-oriented motivational climate.

Given the importance of motivation, anxiety, and emotional aspects in sports performance, and considering the scarcity of studies conducted within low-level sport, the present study established two aims: (a) To describe the type of MC, levels of anxiety, and EI in a sample of footballers playing at a low competitive level; and (b) to analyze the relationships between these variables in order to advance the knowledge base upon which future studies will be developed.

## 2. Materials and Methods

### 2.1. Design and Participants

A cross-sectional study was carried out in order to analyze the association between levels of anxiety, EI, and MC reported by low-level footballers. The sample was composed of 319 young male footballers (16–18 years old) registered in the province of Jaen. Participants competed at the following levels: Youth Honor Division, National, Preferred, First Provincial, and Second Provincial. These levels correspond to the equivalent of college and development leagues outside of Spain. Convenience sampling was used to select the study sample from the aforementioned divisions. Thirty-seven questionnaires were eliminated from the final dataset because they were incompletely or incorrectly completed. Thus, the final sample included 282 registered football players aged between 16 and 18 years old (16.96 ± 0.77).

### 2.2. Measurements

The following instruments were used in the present study:

- State-Trait Anxiety Inventory (STAI). This scale was developed by Spielberger et al. [51] and is used to estimate levels of state anxiety and trait anxiety. This instrument is one of those most universally employed in the context of health and sport [52]. The inventory is evaluated using a Likert scale with four options ranging from 0 (“not at all”) to 3 (“very much”). It is composed of 40 items that describe two categories: State anxiety (anxiety produced at a given time in response to a stimulus that is interpreted as threatening) and trait anxiety (prolonged anxiety that persists over time and whose sufferers are often characterized by tendencies to react anxiously to stimuli that are not usually considered stressful). Items 1 to 20 measure trait anxiety, with responses being summed to produce an overall trait anxiety score. Questions 1, 2, 5, 8, 10, 11, 15, 16, 19, and 20 are negatively framed items, making it necessary to invert these scores before analysis. Items 21 to 40 measure state anxiety, with responses similarly being summed to produce an overall state anxiety score. Questions 21, 26, 27, 30, 33, 36, and 39 are negatively framed, making it also necessary to invert these scores before analysis. Overall scores for trait anxiety and state anxiety range from 0 to 60. Both types of anxiety can then be categorized according to the centiles described in the original version of the measure [51]. In the present study, reliability of the instrument was determined using Cronbach’s alpha, with a value of α = 0.94. For the state anxiety subscale, reliability was determined as α = 0.92, while for trait anxiety a value of α = 0.89 was produced;
- Schutte Self-Report Inventory (SSRI). This was adapted from the original questionnaire developed by Schutte et al. [53], which conceptualizes EI as a unifactorial construct. It was adapted by García-Coll et al. [54] into a multifactorial model that describes the following dimensions: Emotional perception, self-emotional management, hetero-emotional management, and emotional utilization. This instrument is made up of 33 items that evaluate the capacity of individuals to identify, understand, and manage their own and other people’s emotions. The items are evaluated on a five-point Likert scale, where 1 = “Totally disagree” and 5 = “Totally agree”. For the present study, the model proposed by García-Coll et al. [54] was used, in which items 5, 28, and 33 are eliminated. These items were not included, as they have been shown to distort the subsequent data analysis. The instrument utilized in the present study therefore consisted of 30 items. In the study by García-Coll et al. [54], a reliability value of α = 0.91 was established for overall EI. The present study obtained an identical value for reliability;
- Perceived Motivational Climate in Sport Questionnaire (PMCSQ-2). This measure was extracted from the original version described by Newton et al. [55] and was adapted into Spanish by González-Cutre et al. [56]. The instrument consists of 33 items rated on a five-point Likert scale, ranging from 1 = “Totally disagree” to 5 = “Totally agree”. The questionnaire establishes two categories: Task climate, which includes subcategories describing cooperative learning, effort/improvement, and important role; and ego climate, which includes subcategories describing punishment of mistakes, unequal recognition, and rivalry between members. Internal consistency (Cronbach’s alpha) of the instrument in its adaptation into Spanish produced a value of α = 0.90 for ego climate and α = 0.84 for task climate [56]. The present research obtained a value of α = 0.89 for ego climate and α = 0.93 for task climate.

### 2.3. Procedure 

First, a meeting was arranged with the president of the Football Federation of Jaen to request its collaboration and to provide information about the nature of the proposed study. Contact with a number of youth clubs was subsequently initiated by the president following this meeting. Presidents of individual teams were then contacted to inform them about the nature of the study, explain the objectives, and request their collaboration. Visits were then arranged with the clubs to collect the relevant data. A study researcher was always present at these visits in order to answer questions, resolve doubts, and ensure that questionnaires were completed correctly. The inclusion criteria were as follows: (1) Being registered at one of the included clubs at the time of the study; and (2) not having suffered any serious injury that prevented participation in football matches or training in the two months immediately prior to the study commencement. Data collection was carried out during the months of March and April 2017.

Confidentiality of participants was respected at all times throughout the study, and the ethical principles for medical research involving human subjects laid out in the Declaration of Helsinki of 1975 were followed. Informed consent of parents or legal guardians was obtained for all participants. The research Ethics Committee of the University of Granada approved this study with code 641/CEIH/2018.

### 2.4. Data Analysis 

Statistical analysis of the data was performed using the software IBM SPSS version 23.0 (IBM Corp, Armonk, NY, USA). Descriptive analysis was performed using frequencies and medians. Group differences between the described variables were analyzed using ANOVA with Bonferroni adjustment to examine intergroup differences. Pearson’s bivariate correlations were used to examine the relationships between variables. In addition, Kolmogorov–Smirnov’s test with Lillieforts’s correction was used in order to check the normality of the data. Levene’s test was employed in order to check homoscedasticity. Finally, Cronbach’s alpha coefficient was used to analyze the internal reliability of the instruments used, establishing the reliability index at 95.5%.

## 3. Results

As can be seen in Table 1, more than half of the participants surveyed reported low-state anxiety (52.3%; *n* = 150), with 45.4% (*n* = 128) reporting medium levels and only 1.4% (*n* = 4) reporting high state anxiety. With regards to trait anxiety, almost half of participants were found to have medium levels (48.9%; *n* = 138), followed by 48.2% (*n* = 136) reporting a low level and the remaining 2.8% (*n* = 8) reporting high trait anxiety.

In the case of EI, the average score for overall EI was 3.90 ± 0.44. When considering the different dimensions that make up this construct, self-emotional management was observed to be the construct that obtained the highest score, with an average of 4.03 ± 0.42. The next highest score was recorded for emotional perception (3.88 ± 0.51), followed by hetero-emotional management (3.87 ± 0.51), with emotional use being the least valued dimension on average (3.87 ± 0.55). Average values reported for the various dimensions of EI were similar, ranging between 4.03 and 3.78 and showing little variation.

When analyzing the MC perceived by the footballers included in the present sample, it can be seen that a task-oriented climate (3.98 ± 0.74), and its dimensions cooperative learning (4.09 ± 0.70), important role (4.01 ± 0.75), and effort/improvement (3.91 ± 0.63), obtained similar scores. This MC produced higher scores than those reported in relation to an ego-oriented climate (2.38 ± 0.74) and its categories, rivalry between group members (2.81 ± 0.90), unequal recognition (2.31 ± 0.84), and punishment of mistakes (2.25 ± 0.82).

Table 2 presents the analyzed associations between EI and levels of anxiety. Statistically significant differences can be seen (*p* ≤ 0.05) between all relevant dimensions. These differences are explained by the fact that footballers with low levels of anxiety reported the highest scores for overall EI (4.03 ± 0.40) and its associated four dimensions. Moreover, respondents with high levels of state anxiety also reported lower scores for EI (3.61 ± 0.01). The association between overall EI and its four dimensions with trait anxiety showed statistically significant differences at the level *p* ≤ 0.05. Athletes with low anxiety obtained the highest scores for overall EI (4.08 ± 0.40) and its four dimensions, while young people with higher levels of anxiety presented the lowest scores for EI (3.71 ± 0.12).

Table 3 presents the analyzed associations between footballers’ anxiety levels and MC. In terms of state anxiety, statistically significant differences were found (*p* ≤ 0.05) for all categories apart from rivalry between group members. These differences were due to the fact that footballers who presented low anxiety reported perceiving a task-oriented climate (4.16 ± 0.47 vs 3.82 ± 0.40) to a greater extent than their counterparts with high anxiety. Respondents who reported average anxiety levels also perceived an ego-oriented climate (2.53 ± 0.77 vs 1.53 ± 0.03) to a greater extent. In considering trait anxiety, the only statistically significant association found (*p* ≤ 0.05) was between a task-oriented climate and its dimensions effort/improvement and important role. Finally, statistically significant associations regarding an ego-oriented climate were not found. These differences can be explained by findings that footballers reporting low anxiety had higher average values for task climate compared to those who reported high or medium anxiety (4.10 ± 0.42). They also reported higher values for the dimensions effort/improvement (4.06 ± 0.51) and important role (4.22 ± 0.52). There was no statistically significant association between an ego-oriented climate and trait anxiety.

Correlational analysis between EI and task climate (Table 4) showed that EI correlated significantly (*p* ≤ 0.01) with a task-oriented MC. A task-oriented climate was positively related to overall EI (*r* = 0.379) and with all of its related dimensions: Emotional perception (*r* = 0.352), self-emotional management (*r* = 0.343), hetero-emotional management (*r* = 0.320), and emotional utilization (*r* = 0.360). There were positive relationships between overall EI and its dimensions, with the strongest correlation being found with hetero-emotional management (*r* = 0.929) and the lowest score with emotional utilization (*r* = 0.734). Moreover, task-oriented climate was directly associated with its dimensions. The strongest correlation was identified with effort/improvement (*r* = 0.956), followed by cooperative learning and important role, which produced the same value (*r* = 0.927). 

Correlational analysis between EI and ego climate (Table 5) indicated that EI did not correlate with an ego-oriented MC, suggesting that there was no relationship between the EI of athletes and their motivational orientation toward ego perceptions. Furthermore, an ego-oriented climate was directly associated with its dimensions. The strongest correlation was produced for unequal recognition (*r* = 0.951), followed by punishment of mistakes (*r* = 0.924) and member rivalry (*r* = 0.763).

## 4. Discussion

The objective of the present study was to analyze the relationships between MC, anxiety, and EI, as well as to describe these variables in a sample of low-level footballers. Research in this type of population is scarce [57,58,59], although numerous studies of a similar nature have been conducted in the field of highly competitive sport and performance [43,46,60,61].

With regard to the anxiety levels identified in the present sample, more than half of participants experienced only low levels of state anxiety. These data were in accordance with those found in a study by Selmi et al. [12] with a sample of athletes who also reported low levels of state anxiety following involvement in an intervention. In the same sense, research carried out by Zurita-Ortega et al. [62] on a sample of youth football players found more than half of participants to show low state anxiety. The number of participants reporting high values was very low, which was consistent with data obtained by Aguirre-Loaiza and Ramos [63], Martínez-Monteaguado et al. [64], and Abenza et al. [65]. On the other hand, these data did not agree with findings reported by Balyan et al. [66] with a sample of athletes who reported high levels of state anxiety, with high state anxiety in turn being positively related to competitiveness. It is important to highlight that the low number of athletes with a high state anxiety could be explained by the fact that the analyzed sample belonged to lower categories. Most young people practice this sport due to its recreational component in order to enjoy it, which is not linked to high levels of anxiety. Specifically, subjects with high anxiety seek to progress in this sport, trying to reach higher competitive levels, so their levels of anxiety increase [64,65].

With regard to trait anxiety, almost half of participants reported medium levels. In this sense, the study carried out by Zurita-Ortega et al. [62] showed similar data in a group of young athletes. Likewise, the research carried out by Fernández-García et al. [67] on young sportsmen and -women found that more than half of their respondents had medium levels of trait anxiety, with very few reporting low values. In relation to the findings of other studies, these levels of anxiety can be considered to be high. For instance, taking into account the study conducted by Olmedilla et al. [68], in which trait anxiety of young footballers aged between 16 and 19 was very low, the figures reported in the present study were relatively high. Following the previous basis, the levels of high trait anxiety were due to the fact that a small group of respondents interpret the situations that they face in this sport as threatening. This is explained by their interest in achieving professional levels of sport practice in order to increase economic rewards and social recognition [66,68].

When analyzing EI, generally good levels were reported for this psychological construct, highlighting appropriate management and perception of emotions, although the findings suggest that emotional use needs improvement. Along the same lines, Akelaitis and Malinauskas [43] found adequate levels of EI in a sample of young athletes from both individual and collective sports. Similarly, their study corroborated the present findings that management was the dimension perceived most strongly by young athletes. Similar data were reported in a study conducted by Castro-Sánchez et al. [69], in which participants who practiced team sports reported higher values for emotional management than for the other EI dimensions. Further findings coming from investigation of the separate dimensions were those reported by Bekendam [70] and Pérez [71], who found emotional use to be significantly lower than the other dimensions. In contrast, in the thesis developed by Lezcano [72] with a sample of young footballers, emotional use was found to be greater than management. 

In examining the MC of the included football players, it can be observed that the present study tended to orient more toward a task climate than to an ego-oriented climate. Cooperative learning was the dimension that most determined a mastery environment, whereas rivalry between members was the most influential factor when the environment focused on performance. In this sense, the studies developed by Galván et al. [73] and Jaakkola et al. [74] found young athletes tended to orient more toward a task climate and demonstrated a strong predisposition toward cooperative learning and personal improvement in their performance, while at the same time reporting high levels of rivalry and concern for the final outcome [75]. Further work carried out by Del Castillo-Andrés et al. [76] showed that male athletes tended to orientate toward an ego climate and indicated that they desired to demonstrate superiority in relation to physical abilities.

In analyzing the relationship between EI and state anxiety, it is seen that footballers with low levels of anxiety also demonstrated higher overall EI, while low levels of this emotional factor were related to a higher state of anxiety. The present findings were similar to those obtained by Ros-Martínez et al. [77], who stated that emotional skills within the sports environment are essential as they contribute to improved emotional control, avoiding responses such as stress, anxiety, or anger. As has been noted by Webster et al. [78], levels of EI are higher in collective sports due to the regular interactions between players and the sharing of pressure within all members of the collective, meaning that anxiety and rash responses are reduced.

The association between EI and trait anxiety showed a similar pattern, in that athletes with low anxiety also tended to report higher EI, while young people with more symptoms of anxiety had a lower capacity for emotional regulation. Along the same lines, the structural equation model developed by Castro-Sánchez et al. [48] within a sample of young athletes revealed that EI plays a fundamental role in achieving optimum levels of sport performance. This was attributed to the development of a positive regulation and use of emotions by participants, leading to greater mental well-being and lower trait anxiety. These findings have also been reiterated by Kuan et al. [79].

When comparing anxiety to motivational aspects, it is necessary to highlight relationships between levels of anxiety and the types of MC. Young footballers who presented low levels of anxiety were oriented toward a climate of mastery, while those who experienced medium or high levels of anxiety opted for performance-focused environments. These findings were similar to those reported by the majority of studies that have previously been conducted within the sports field [18,80,81,82]. It is therefore accepted that when athletes’ perceptions of an environment foregrounds task-related aspects, low or medium levels of anxiety result [21,33,48,59], while a MC that centers on performance aspects can relate to high anxiety levels [61,83]. As for the relationships regarding trait anxiety, it is evident that participants presenting with low levels of anxiety report higher values on average for a task climate in comparison to those reporting high or medium levels of anxiety [31,84]. In this sense, athletes oriented toward mastery experience a better state of anxiety because they focus on personal improvement and the inherent benefit of the athletic activity. In other words, they focus attention on intrinsic factors. On the other hand, those who are oriented toward an ego environment focus on extrinsic factors that are often out of their control, causing situations of stress and a concomitant increase in anxiety [85,86].

In addition, it should be noted that EI and a task-oriented climate were positively related. This was due to the role of perception and use of emotions, which are key factors in identifying the benefits of engaging in sport, as well-being is key to experiencing satisfaction when participating in sporting disciplines such as football. Similarly, it has been reported that control of motivation and the autonomous motivation of athletes are related to emotional control and regulation [40]. Further, when autonomous motives for participation are satisfied, it is more likely that young individuals will effectively develop their understanding and regulation of emotional aspects [87,88]. When considering the relationship that exists between the educational environment and sporting experiences at low-level sport engagement, it should be highlighted that positive emotions and intrinsic motivation are interrelated, with motivation based on personal satisfaction producing positive emotional levels [89]. On the other hand, the present study suggested that there was no relationship between the EI of athletes and ego-oriented motivations. This idea has been corroborated in other studies, such as that conducted by Campo et al. [90], who identified that EI could be improved in the absence of preexisting extrinsic motivations. 

Finally, it is important to highlight some limitations of the present study. The first of these lay in the design, which was descriptive and cross-sectional. This methodological design was useful for understanding the state of the problem and provided preliminary evidence of the relationships between anxiety, EI, and MC. However, causal relationships could not be established. Second, the study sample employed should be highlighted. Although the number of participants was high, it would have been interesting and informative to have conducted random sampling stratified according to the different zones of the province of Jaen. Moreover, it would be interesting to replicate this study while expanding the number of respondents and including other variables such as period of the season, injuries suffered, and performance outcomes. In addition, future studies are encouraged to develop an intervention program in order to verify the combined effect of EI and task-oriented climates on the anxiety levels of young footballers.

## 5. Implications for Practice

The present research provides relevant data about the relationship between anxiety, EI, and MC in young footballers participating within the lower tiers of the sport. It provides a basis from which future studies may be able to develop intervention programs, targeting reductions in the anxiety felt by athletes via motivational strategies and emotional techniques. Specifically, the present findings indicate that participants oriented toward a task climate and with a better capacity to control their emotions tend to show lower levels of anxiety. 

The present study employed a cross-sectional design, which does not allow definitive recommendations to be made regarding the development of treatment programs. Nevertheless, special attention should be paid to the existing relationships between high levels of anxiety, low EI, and an ego-oriented climate. Thus, the role of coaches working with athletes in low-tier sport should be considered. Coaches should be encouraged to promote a task-oriented motivational climate in order to feed intrinsic motivations during the competitive period and to avoid high levels of anxiety. This can be achieved through the use of communicative tools, teamwork, tasks for raising self-efficacy, and interrogative feedback, all of which favor the compartmentalization and regulation of emotions. In addition, anxiety levels of athletes should be monitored during the competitive period in order to improve subsequent performance. 

## 6. Conclusions

Considering the research question that was presented, the existence of a negative relationship between anxiety, emotional intelligence, and task-oriented motivational climate can be confirmed. In contrast, an ego-oriented motivational climate was positively related to both state and trait anxiety. The following conclusions can therefore be made in relation to the initial hypotheses:

- Hypothesis 1 (H1) was fulfilled, given that footballers reporting lower levels of state anxiety and trait anxiety also understood and regulated their emotions better;
- Hypothesis 2 (H2) was partially fulfilled. Footballers with lower levels of anxiety also reported higher scores in relation to a task-oriented motivational climate and its dimensions. Nevertheless, those who reported greater perceptions of an ego climate were also more likely to present medium levels of anxiety;
- Hypothesis 3 (H3) was fulfilled, since the dimensions of emotional intelligence were positively related to the dimensions of a task climate. This highlights the positive effect of these psychosocial factors on the anxiety of football players.

The present study presents as its main conclusions that young footballers from lower tiers showed high levels of state and trait anxiety, with at least half of the present sample providing high scores. With regard to overall EI, adequate levels were reported, suggesting that management and perception of emotions was appropriate, although emotional use requires improvement. In addition, the included footballers were more oriented toward task (mastery) than toward ego (performance), emphasizing cooperative learning in mastery environments and little rivalry between members in performance contexts.

Athletes with the highest EI had low levels of state and trait anxiety, while young people with greater anxiety were unable to adequately use and regulate their emotions. Likewise, players who were oriented more strongly toward a task climate showed lower levels of anxiety relative to those who were oriented toward environments linked to performance.

Finally, there was a positive relationship between EI and a task-oriented climate, given that the perception and use of emotions are key factors in identifying the benefits of sports practice, as well as in satisfaction itself. On the other hand, it can be concluded from the present study that no relationship emerged between EI and motivational orientation toward the ego within young footballers. 

## Figures and Tables

**Table 1:** Anxiety levels, emotional intelligence, and motivational climate.

**State Anxiety**	
Low	52.3% (*n* = 150)
Average	45.4% (*n* = 128)
High	1.4% (*n* = 4)
**Trait Anxiety**	
Low	48.2% (*n* = 136)
Average	48.9% (*n* = 138)
High	2.8% (*n* = 8)
**Emotional Intelligence**	**M ± SD**
Emotional intelligence	3.90 ± 0.44
Emotional perception	3.88 ± 0.51
Auto-emotional management	4.03 ± 0.42
Hetero-emotional management	3.87 ± 0.51
Emotional use	3.78 ± 0.55
**Motivational Climate**	**M ± SD**
Ego climate	2.38 ± 0.74
Punishment of mistakes	2.25 ± 0.82
Unequal recognition	2.31 ± 0.84
Intrateam member rivalry	2.81 ± 0.90
Task climate	3.98 ± 0.64
Cooperative learning	4.09 ± 0.70
Effort/improvement	3.91 ± 0.63
Important role	4.01 ± 0.75

**Table 2:** Association between anxiety levels and emotional intelligence.

**Dimensions for Emotional Intelligence**	**State Anxiety**			***F***	**Sig.**
	**High (*n* = 4)**	**Medium (*n* = 128)**	**Low (*n* = 150)**		
	**M ± SD**	**M ± SD**	**M ± SD**		
Emotional intelligence	3.61 ± 0.01	3.76 ± 0.45	4.03 ± 0.40 *	14.89	0.000
Emotional perception	3.50 ± 0.14	3.73 ± 0.49	4.02 ± 0.48 *	13.95	0.000
Auto-emotional management	3.75 ± 0.00	3.91 ± 0.45	4.15 ± 0.37 *	13.03	0.000
Hetero-emotional management	3.40 ± 0.19	3.73 ± 0.54	3.99 ± 0.45 *	11.29	0.000
Emotional use	3.62 ± 0.56	3.910.50	4.12 ± 0.43 *	11.11	0.000
**Dimensions for Emotional Intelligence**	**Trait Anxiety**			***F***	**Sig.**
	**High (*n* = 8)**	**Medium (*n* = 138)**	Low (*n* = 136)		
	**M ± SD**	**M ± SD**	M ± SD		
Emotional intelligence	3.71 ± 0.12 **	3.74 ± 0.42	4.08 ± 0.40 *	22.92	0.000
Emotional perception	3.56 ± 0.11 **	3.70 ± 0.49	4.09 ± 0.46 *	25.18	0.000
Auto-emotional management	3.87 ± 0.16	3.90 ± 0.43	4.18 ± 0.37 *	18.13	0.000
Hetero-emotional management	3.57 ± 0.19 **	3.69 ± 0.50	4.06 ± 0.46 *	21.96	0.000
Emotional use	3.67 ± 0.51	3.87 ± 0.57	4.06 ± 0.47 *	5.91	0.003

Note: SD = standard deviation; * = significantly different from the medium-anxiety group; ** = significantly different from the low-anxiety group. *F*: F-test.

**Table 3:** Association between anxiety levels and motivational climate.

**Dimensions for Motivational Climate**	**State Anxiety**			***F***	**Sig.**
	**High (*n* = 4)**	**Medium (*n* = 128)**	**Low (*n* = 150)**		
	**M ± SD**	**M ± SD**	**M ± SD**		
Ego climate	1.53 ± 0.03 *	2.53 ± 0.77	2.28 ± 0.69 *	7.02	0.001
Punishment of mistakes	1.50 ± 0.00	2.46 ± 0.87	2.10 ± 0.74 *	8.72	0.000
Unequal recognition	1.28 ± 0.16 *	2.46 ± 0.85	2.21 ± 0.82	6.19	0.002
Intrateam member rivalry	2.16 ± 0.19	2.85 ± 0.86	2.80 ± 0.93	1.16	0.313
Task climate	3.82 ± 0.40	3.78 ± 0.76	4.16 ± 0.47 *	12.37	0.000
Cooperative learning	3.87 ± 0.43	3.88 ± 0.78	4.27 ± 0.57 *	11.30	0.000
Effort/Improvement	3.75 ± 0.28	3.74 ± 0.73	4.06 ± 0.51 *	9.23	0.000
Important role	3.90 ± 0.57	3.78 ± 0.91	4.22 ± 0.52 *	12.83	0.000
**Dimensions for Motivational Climate**	**Trait Anxiety**			***F***	**Sig.**
	**High (*n* = 8)**	**Medium (*n* = 138)**	**Low (*n* = 136)**		
	**M ± SD**	**M ± SD**	**M ± SD**		
Ego climate	2.20 ± 0.75	2.46 ± 0.67	2.31 ± 0.80	1.72	0.181
Punishment of mistakes	2.25 ± 0.88	2.37 ± 0.75	2.14 ± 0.87	2.75	0.066
Unequal recognition	2.10 ± 1.06	2.36 ± 0.76	2.26 ± 0.91	0.73	0.483
Intra-team member rivalry	2.33 ± 0.90	2.88 ± 0.87	2.77 ± 0.91	1.75	0.175
Task climate	3.87 ± 0.71	4.09 ± 0.56	4.10 ± 0.42 *	4.49	0.012
Cooperative learning	3.99 ± 0.74	4.17 ± 0.65	4.25 ± 0.50	2.50	0.084
Effort/Improvement	3.81 ± 0.71	4.00 ± 0.55	4.09 ± 0.41 *	3.34	0.037
Important role	3.85 ± 0.81	4.00 ± 0.65	4.18 ± 0.66 *	6.77	0.001

Note: SD = standard deviation; * = significantly different from the medium-anxiety group; ** = significantly different from the low-anxiety group. *F*: F-test.

**Table 4:** Correlation between task climate and emotional intelligence.

Dimensions	EP	SEM	HEM	EU	TC	TCCL	TCEI	TCIR
EI	0.925 ^*^	0.917 ^*^	0.929 ^*^	0.734 ^*^	0.379 ^*^	0.355 ^*^	0.343 ^*^	0.375 ^*^
EP	-	0.812 ^*^	0.796 ^*^	0.612 ^*^	0.352 ^*^	0.335 ^*^	0.318 ^*^	0.344 ^*^
SEM	-	-	0.797 ^*^	0.624 ^*^	0.343 ^*^	0.333 ^*^	0.307 ^*^	0.335 ^*^
HEM	-	-	-	0.569 ^*^	0.320 ^*^	0.298 ^*^	0.292 ^*^	0.314 ^*^
EU	-	-	-	-	0.360 ^*^	0.313 ^*^	0.326 ^*^	0.374 ^*^
TC	-	-	-	-	-	0.927 ^*^	0.956 ^*^	0.927 ^*^
TCCL	-	-	-	-	-	-	0.840 ^*^	0.818 ^*^
TCEI	-	-	-	-	-	-	-	0.805 ^*^

Note: * *p* ≤ 0.01; EI = emotional intelligence; EP = emotion perception; SEM = self-emotional management; HEM = hetero-emotional management; EU = emotional utilization; TC = task climate; TCCL = cooperative learning; TCEI = effort/improvement; TCIR = important role.

**Table 5:** Correlation between ego climate and emotional intelligence.

Dimensions	EP	SEM	HEM	EU	EC	ECMR	ECPM	ECUR
EI	0.925 ^*^	0.917 ^*^	0.929 ^*^	0.734 ^*^	−0.048	0.020	−0.079	−0.047
EP	-	0.812 ^*^	0.796 ^*^	0.612 ^*^	−0.057	0.038	−0.071	−0.056
SEM	-	-	0.797 ^*^	0.624 ^*^	−0.056	−0.013	−0.084	−0.034
HEM	-	-	-	0.569 ^*^	−0.011	0.052	−0.039	−0.017
EU	-	-	-	-	−0.079	−0.061	−0.079	−0.050
EC	-	-	-	-	-	0.763 ^*^	0.924 ^*^	0.951 ^*^
ECRMG	-	-	-	-	-	-	0.584 ^*^	0.612 ^*^
ECPM	-	-	-	-	-	-	-	0.779 ^*^

Note: * *p* ≤ 0.01; EI= emotional intelligence; EP = emotion perception; SEM = self-emotional management; HEM = hetero-emotional management; EU = emotion use; EC = ego climate; ECMR = intrateam member rivalry; ECPM = punishment of mistakes; ECUR = unequal recognition.
